# RTK-GNSS Increment Prediction with a Complementary “RTK-SeqNet” Network: Exploring Hybridization with State-Space Systems

**DOI:** 10.3390/s25206349

**Published:** 2025-10-14

**Authors:** Hassan Ali, Malik Muhammad Waqar, Ruihan Ma, Sang Cheol Kim, Yujun Baek, Jongrin Kim, Haksung Lee

**Affiliations:** 1 Department of Electronics and Information Engineering, Jeonbuk National University, Jeonju 54896, Republic of Korea; 202350496@jbnu.ac.kr (H.A.); malikwaqarhaider@jbnu.ac.kr (M.M.W.); maruihan@jbnu.ac.kr (R.M.); 2 Core Research Institute of Intelligent Robots, Jeonbuk National University, Jeonju 54896, Republic of Korea; 3 Intelligent Robot Studio Inc., Suwon 16677, Republic of Korea; bak3@paran.com (Y.B.); help@k-irs.com (J.K.); 4 National Institute of Crop and Food Science, Wanju 55365, Republic of Korea; lhs0221@korea.kr

**Keywords:** deep learning, Gated Recurrent Units, Real-Time Kinematic, Inertial Measurement Unit, RTK-GNSS increment prediction, GNSS outages

## Abstract

Accurate and reliable localization is crucial for autonomous systems operating in dynamic and semi-structured environments, such as precision agriculture and outdoor robotics. Advances in Global Navigation Satellite System (GNSS) technologies, particularly Differential GPS (DGPS) and Real-Time Kinematic (RTK) positioning, have significantly enhanced position estimation precision, achieving centimeter-level accuracy. However, GNSS-based localization continues to encounter inherent limitations due to signal degradation and intermittent data loss, known as GNSS outages. This paper proposes a novel complementary RTK-like position increment prediction model with the purpose of mitigating challenges posed by GNSS outages and RTK signal discontinuities. This model can be integrated with a Dual Extended Kalman Filter (Dual EKF) sensor fusion framework, widely utilized in robotic navigation. The proposed model uses time-synchronized inertial measurement data combined with the velocity inputs to predict GNSS position increments during periods of outages and RTK disengagement, effectively substituting for missing GNSS measurements. The model demonstrates high accuracy, as the total aDTW across 180 s trajectories averages at 1.6 m while the RMSE averages at 3.4 m. The 30 s test shows errors below 30 cm. We leave the actual Dual EKF fusion to future work, and here, we evaluate the standalone deep network.

## 1. Introduction

Accurate and reliable localization is crucial for autonomous systems operating in dynamic and semi-structured environments, such as precision agriculture and outdoor robotics. Advances in Global Navigation Satellite System (GNSS) technologies, particularly Differential GPS (DGPS) and Real-Time Kinematic (RTK) [1] positioning, have significantly enhanced position estimation precision, achieving centimeter-level accuracy. These advancements have facilitated notable progress in applications that require precise navigation and mapping.

However, GNSS-based localization continues to encounter inherent limitations due to signal degradation and intermittent data loss [2], known as GNSS outages. Such outages commonly occur in environments characterized by limited satellite visibility, including dense forests, urban canyons, remote regions, or areas surrounded by tall structures, adversely impacting localization accuracy and system reliability [3]. Traditional sensor fusion methods, such as the Dual Extended Kalman Filter [4] (Dual EKF), effectively fuse GNSS data with Inertial Measurement Units (IMUs) to enhance positioning accuracy and robustness. Nevertheless, these approaches exhibit limitations when GNSS signals become unavailable for extended periods, leading to drift accumulation and significant positional errors.

Motivated by these limitations and the growing demand for reliable, autonomous navigation in challenging environments, this paper introduces a novel complementary GNSS increment prediction model for hybridization with the Dual EKF framework. Employing advanced temporal sequence modeling techniques, including convolutional layers and bi-directional Gated Recurrent Units [5] (GRUs), the model predicts GNSS increments using historical inertial measurements, acceleration data, and temporal context. This approach effectively mitigates the effects of missing GNSS data during RTK outages, ensuring robust, continuous, and accurate localization.

The proposed method significantly enhances system resilience by providing reliable position corrections when GNSS signals are unreliable or unavailable. Consequently, the model ensures consistent and precise localization performance, contributing substantially to advancing autonomous navigation in real-world, adverse conditions. This paper proposes a many-to-many GRU-based architecture “RTK-SeqNet” that uses a novel composite loss approach, which exploits the multiscale temporal features from the model to scrutinize the increments between adjacent positions, a direction loss that calculates the angle with respect to the horizontal axis, with a higher priority given to the recent prediction and a Vector Loss calculating the magnitude and direction of each prediction individually, such that each increment spacing and the relative angle between the contiguous vectors are learned for coherence in the predicted trajectory. The many-to-many aspect allows multiple predictions for a single point that can be integrated to minimize the effect of historical erroneous predictions.

The remainder of this paper is organized as follows. In Section 2, the effectiveness of predicting the incremental position by using inertial data and its viability is established, in the Choice of GRU over LSTM Section, the comparison and choice of GRUs over Long Short-term Memory units (LSTMs) is discussed along with the previous works using these techniques. In Section 3, Section 3.1 discusses the data preparation techniques, Section 3.2 explores the proposed RTK-SeqNet architecture in detail, and Section 3.3 discusses the composite loss function and optimization parameters. In Section 4, the detailed results of the model are presented, where Section 4.1 shows results for the Vector Loss based direction learning progression, and Section 4.2 shows the results of model performance using DTW and RMSE. Finally, Section 5 and Section 6 discuss the future work combining the Dual Extended Kalman Filter approach with the proposed model and the Conclusion, respectively.

## 2. Related Works

Most recent works on this topic employ AI-based methods to rectify this problem; this is achieved by either predicting the error between the GNSS and inertial dead reckoning-based positioning $\left(O_{INS}-\delta P_{INS}\right)$ where $\delta P_{INS}$ denotes the inertial dead reckoning position error with respect to the GNSS-based location and $O_{INS}$ is the dead reckoning-based INS position. $X_{K}$ is the state vector that represents the current position and orientation of the rover, and by taking $O_{INS}$ as the input, $X_{K}$ can be directly predicted i.e., ${(O}_{INS}-X_{k}),$ and lastly, $\left(O_{INS}-∆P\right)$ predicts the GNSS increments directly in terms of ∆northings and ∆eastings combined as ∆P by taking $O_{INS}$ as the input. Predicting the increments in relation to the inertial position promises minimal mixing errors as compared to the former two methods [6]. This paper uses the ${(O}_{INS}-∆P)$ approach with slight changes, as discussed in the following section. The GNSS position equation is given by

(1) ∆PGNSS=∫∫vn^ (t)dtdt=∫∫Cbnfibb t−2wien t+wenn t×vnt+Gndtdt

The equation converts the velocity in the navigation frame ($v_{n}$) to the GNSS position. The specific force $f_{ib}^{b}$ (i.e., specific force measured in the body frame and relative to the inertial frame while existing in the body frame) is measured by the IMU in the body frame, which is then projected into the navigation frame by the transformation matrix $C_{b}^{n}$ (i.e., transformation from the rover’s perspective to the lofty perspective of the navigation frame). The Coriolis effect [7] due to Earth’s rotation can be represented by $2w_{ie}^{n}(t)$, accounting for any change or deflection in the apparent movement of the rover due to Earth’s rotation; similarly, to account for the relative angular velocity between the Earth’s frame and the navigation frame and the rotation of the navigation frame itself as the rover traverses the curved surface of the Earth, $w_{en}^{n}(t)$ (transport effect) is added to the Coriolis term. Lastly, $G_{n}$ accounts for the gravitational acceleration in the navigation frame, which was not included in the specific force $f_{ib}^{b}$. This model for the prediction of GNSS increments only requires the INS inputs for the current time step.

### Choice of GRU over LSTM

Vanilla Recurrent Neural Networks [8], while giving satisfactory results for short sequences, do not scale well for tasks that require an ability of long-term information retention due to their limited memory capacity. Standard RNNs also face the problem of vanishing or exploding gradients, which is the corollary of their limited learning capacity, making the learning unstable and a subsequent need for careful weights initialization.

The aforementioned problems are solved in both the LSTM [9] and GRU architecture; however, they have nuances that may make them more or less suitable for certain tasks. The foremost distinction between LSTMs and GRUs is that the latter has a simpler gating structure with only two gates, whereas the former has three gates. Table 1 lists each gate and its role in either type of RNN unit.

Courtesy of having one less control gate, GRUs have less trainable parameters than equivalent LSTMs. This reduced complexity mitigates overfitting and lowers memory usage. A direct advantage of lowering complexity and memory usage is that the model becomes more efficient and relatively less expensive in terms of computation; this is desirable for real-time applications, especially when other algorithms and processes are being run simultaneously by the same computing resource. Table 2 compares the number of parameters and time required by the RNN units in the inference mode; the GPU device used was an Nvidia RTX 3050 (manufactured in Samsung Electronics, Hwaseong-si, South Korea) with 6 GB of memory. In terms of performance, despite being simpler, the GRU networks retain accuracy. In an experiment conducted by Yang et al. where real-time trajectory is predicted using GRUs for small-scale quadcopters, the model predicted real-time trajectory data, which was on par with the trajectory predicted offline (after post-processing) [10]. Hu et al. present a model-assisted navigation system for an autonomous tractor that uses a CNN-BiLSTM network to learn the vehicle’s motion patterns under normal RTK-GNSS reception and then predict the vehicle’s positions when GNSS signals are denied [11]. In a 100 s GNSS outage test, their proposed model showed an average position error nearly 79% lower as compared to the simple INS. Hareth et al., proposed an inertial navigation method RoNIN which uses 1D ResNet CNN (and variants with LSTM and TCN) to regress velocity vectors directly from IMU time-series data. This method outperforms previous RNN-based approaches [12]. Cao et al. proposed a PSO-LSTM model to enhance the GNSS/INS navigation when satellite data is not available. This approach utilizes an LSTM network to predict increments using only the historical positions and IMU data. This approach, in a 60 s road test, saw a cut in INS drift up to 99% [13]. Finally, Lin et al. proposed an end-to-end deep sensor fusion model for localization. It uses MotionNet for learning motion dynamics from IMU and wheel odometry, a MeasurementNet which is an LSTM-based network for gauging satellite data quality. Finally, both the streams are fused using an RNN network to estimate the final state [14]. Another approach is to combine RNN model predictions with sensor fusion to improve reliability. Zhao et al. propose an LSTM model that generates “pseudo-GNSS” measurements during urban signal losses [15].

Compared to recent state-of-the-art studies, our proposed RTK-SeqNet introduces several methodological innovations that distinctly enhance GNSS outage prediction accuracy. While approaches such as those by Yang et al. and Cao et al. primarily rely on simpler recurrent architectures (GRU or LSTM alone), our method integrates hierarchical multiscale convolutional structures with bi-directional GRUs, effectively capturing both local nuances and global contextual patterns. Unlike Hu et al., who employed standard CNN-BiLSTM networks, RTK-SeqNet explicitly incorporates a structured vector-learning framework to ensure directional and incremental coherence, crucial for long-term trajectory accuracy. Furthermore, our composite loss function comprising GNSS loss, directional loss, and Vector Loss provides targeted constraints, systematically addressing issues related to cumulative drift and directional inaccuracies common in simpler scalar-loss methods used in referenced studies. Lastly, while earlier approaches typically adopt one-to-many or basic incremental strategies, our many-to-many integration approach iteratively averages overlapping predictions, significantly reducing accumulated errors over extended GNSS signal interruptions. Collectively, these enhancements position RTK-SeqNet as a robust and highly accurate method, offering substantial improvements over existing approaches in handling GNSS outages effectively.

## 3. Data Construction and Proposed Method

### 3.1. Data Collection and Processing

Having established the effectiveness of predicting $∆P_{GNSS}$ for the future trajectory, the data preparation for training is performed in two steps: (1) raw data acquisition from all sensors with noise removal algorithms; (2) post-processing step for pruning the collected data and synchronizing time steps of sensor data collected at different rates. The data collection process utilizes ROS (Melodic) nodes simultaneously reading sensor data. The raw sensor readings were passed directly to an Extended Kalman Filter and Complementary Filter to mitigate errors such as gyroscope drift and magnetometer biases. Data were structured into standard ROS message types, including odometry (recording positional and orientation changes), Twist (capturing positional and orientation input), and Imu (providing orientation changes, angular velocity, and linear acceleration).

This structured approach inherently provided timestamps, facilitating subsequent time-based filtering. To ensure accuracy and consistency, linear acceleration data was filtered using an EKF instance to minimize noise, complementing corresponding Twist messages. Notably, acceleration data required additional attention due to the susceptibility to noise from vibrations and abrupt movements.

The training dataset was collected using a WeGo Robotics Scout 2.0 mobile platform equipped with an RTK2U GNSS module and a Pololu UM7-LT IMU. The rover was driven along a 10 min unconstrained trajectory designed to capture a wide range of motion dynamics, including variable speeds, rotational maneuvers, and sub-trajectories of diverse geometric patterns to promote model generalization. For evaluation, a separate test trajectory was executed following a more structured and plausible path characterized by gradual changes in speed and direction, forming an overall spiral-shaped pattern. All data were collected in an open field near Jeonbuk National University, Jeonju, South Korea, under clear GNSS reception conditions.

Data were stored using the ROS 1 (Melodic) package rosbag in Ubuntu 18.04 [16], creating binary files. These files inherently recorded timestamps along with data, allowing replay functionality for further validation and precise synchronization. GNSS and IMU data synchronization required timestamps within a threshold of 500 milliseconds; data exceeding this interval were discarded to maintain integrity. After initialization of placeholder arrays for each sensor data, each bag-file entry or “row” is passed through two sets of for-if blocks. These blocks are nested, whereas the first block caters only to the IMU data and the second to GPS data. These blocks are responsible for ensuring that the incoming bag-file entry does have large timestamp discrepancies within sensor values (i.e., among acceleration, gyroscope and NMEA message, VTG message, etc.). Having vetted a particular entry, it is compared with the last entry’s timestamp and discarded if the difference between them exceeds 500 ms.

**Figure 1:**
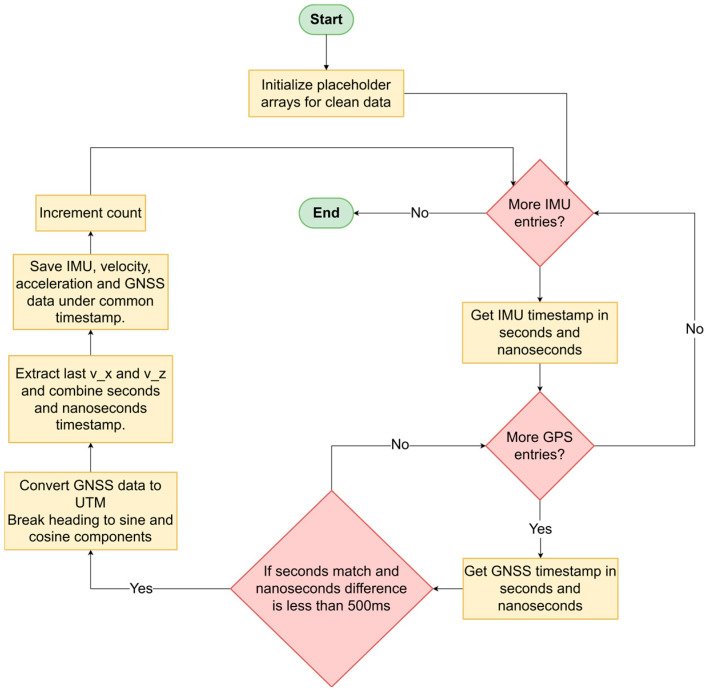
Algorithm for data retention in the post-processing step after data acquisition. All sensor data is synced to within the 500 ms range.

If a row passes these criteria, the data is extracted from it, as shown in Figure 1, after which the process is repeated if there are more bag-file entries available. Addressing cyclic parameters in training models, the heading angles were decomposed into sine and cosine components to mitigate wrap-around issues, bounding angular values within [−1, 1]. Furthermore, GNSS coordinates were converted from the Geographic Coordinate System to the Universal Transverse Mercator (UTM) coordinate system, providing practical metric units conducive to precise trajectory modeling and analysis.

The experiments were conducted on a Scout 2.0 Rover by WeGo Robotics with a maximum linear speed of 1.5 m/s and angular speed of 0.5235 rad/s. The sensors used for this experiment and their specifications are listed in Table 3.

### 3.2. Proposed RTK-SeqNet Architecture

GNSS-based navigation often involves data acquired at varying frequencies and irregular intervals, introducing significant temporal variability and non-linearity into position prediction tasks. Traditional Markov-based [17] models are not robust enough for such conditions: excessively small time steps might fail to capture meaningful state transitions, while excessively large intervals lose critical system dynamics by relying solely on the last known state. Consequently, a novel many-to-many time-series modeling approach was developed, utilizing a hierarchical convolutional and recurrent neural architecture to predict position increments from a known starting point effectively.

**Figure 2:**
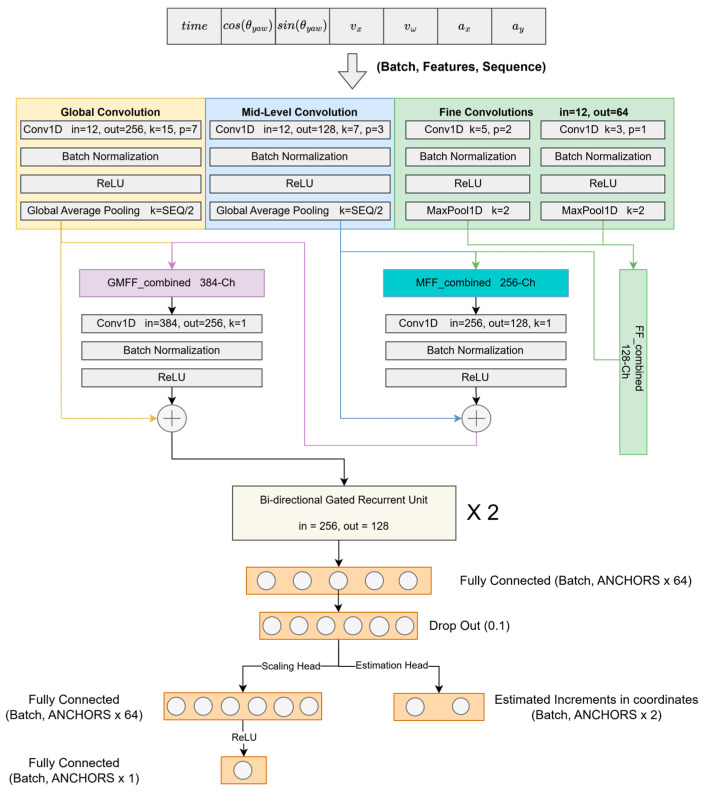
Overall architecture of the RTK-SeqNet Model.

The proposed model architecture, as shown in Figure 2, integrates convolutional and recurrent neural networks to leverage both spatial feature extraction capabilities and temporal dependency modeling. The hierarchical convolutional structures within the proposed model are specifically designed to capture varying ranges of contextual dependencies. The Global Convolution layer, featuring a large kernel (*k* = 15) and pooling size (*p* = 7), effectively identifies long-range dependencies and broader contextual relationships from the input data. This layer is complemented by batch normalization, ReLU activation, and global average pooling to ensure robust feature extraction. The Mid-Level Convolution employs a medium kernel size (*k* = 7) and pooling size (*p* = 3), capturing intermediate-range patterns and refining the features through batch normalization, ReLU activation, and global average pooling operations. Finally, Fine Convolution consists of two parallel layers with smaller kernels (*k* = 5 and *k* = 3) and pooling sizes (*p* = 1 and *p* = 2), specifically targeting short-range dependencies to capture detailed local dynamics. This fine convolutional component also includes batch normalization, ReLU activation, and max pooling operations, further enhancing the granularity and precision of feature representation.

The combined convolutional outputs feed into three distinct feature combination modules (GMFF_combined, MFF_combined, and FF_combined), where further convolution, normalization, and activation refine these features. These processed features then feed into a Bi-directional Gated Recurrent Unit (Bi-GRU) that robustly captures temporal dependencies and irregularities in GNSS sequential data by modeling both past and future contexts simultaneously.

**Figure 3:**
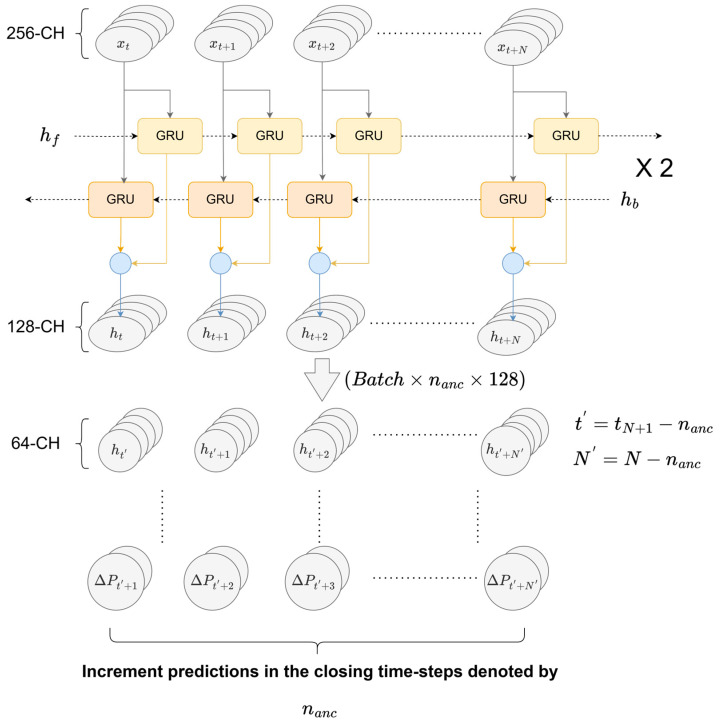
Detailed working of the bi-directional GRU network and the following Fully Connected Layers. The final layer directly predicts the offset of each time step and the corresponding anchor scale factors with respect to the first time step of the sequence.

The model predicts incremental movements or “anchor points” from the last known position of the rover. These anchor points, acting as directional vectors, embody crucial information regarding both the magnitude and direction toward subsequent positions; the detailed anchor point generation from the GRU block output can be seen in Figure 3. As the sequence batch window slides incrementally, certain points are predicted multiple times, and these overlapping predictions are averaged (N_anc times), improving prediction stability and accuracy. The rationale for using anchor points is that the model explicitly forecasts increments from the rover’s last known position. Each anchor point provides critical directional and magnitude information, effectively capturing the evolving direction changes and the incremental growth of positional distances as the rover moves forward in time.

To further refine the prediction capability of the model, two specialized output heads are incorporated: the Scaling Head and the Estimation Head. The Scaling Head is responsible for predicting a scaling factor for each individual predicted increment, producing an output of dimensions (N_anc × 1). This scaling factor enables the model to adaptively adjust the magnitude of each predicted motion increment based on the dynamic intensity of the rover’s movement at each time step. By applying these scaling factors, the model enhances its flexibility and responsiveness to variations in the motion patterns captured within the input data. Importantly, a ReLU activation function is applied to the scaling factors to ensure that they remain non-negative, thereby preserving the physical plausibility of the predicted motion magnitudes. Complementing this, the Estimation Head directly predicts the GNSS positional increments in Universal Transverse Mercator (UTM) coordinates, generating an output of dimensions (N_anc × 2) which correspond to the predicted changes in northing and easting at each time step. These predicted increments represent the robot’s estimated positional changes from its last known location, providing precise spatial updates for navigation purposes. Together, the Scaling Head and Estimation Head enable the model to produce nuanced, dynamically scaled positional predictions that account for both the direction and intensity of the rover’s movement across time.

Data handling involves preprocessing input sequences to accommodate variable data acquisition intervals, maintaining robustness against temporal irregularities. Predictions are incrementally generated and systematically averaged across overlapping predictions to enhance temporal consistency and minimize prediction errors.

### 3.3. Optimization and Loss Functions

Since the model architecture already combines the features at different scales, it is much more useful to have a longer sequence length. The following table shows the hyper-parameter settings.

The weight decay is set to a relatively large value to avoid overfitting the training data, which, despite having high variance, cannot capture all the different conditions that may present themselves. The parameters are summarized in Table 4.

To train the proposed architecture, a composite objective function is designed that simultaneously constrains the absolute position, heading evolution, and vector-field consistency. Formally, the total loss is a weighted linear combination of three complementary terms. Each term targets a distinct failure mode that arises when GNSS observations are absent for extended periods.

(2) $$Loss_{total}=\omega_{GNSS}\;Loss_{GNSS}+\omega_{dir}\;Loss_{Direction}+\omega_{vec}\;Loss_{Vector}$$

where $\omega_{GNSS}$, $\omega_{dir}$, and $\omega_{vec}$ are the weight parameters associated with GNSS increment loss, directional loss, and Vector Loss, respectively. Extensive ablation experiments involving different weight combinations revealed the best working combination to be $\omega_{GNSS}=0.34$, $\omega_{dir}=0.44,$ and $\omega_{vec}=0.22$. As discussed in Section 3.3.2, $Loss_{Direction}$ focuses on the immediate or most recent bearing information, followed by the ${Loss}_{GNSS}$ that tends to the increment loss, and lastly the $Loss_{vector}$ that calculates the inter-point error and directional coherence.

#### 3.3.1. GNSS Loss

During normal operation, centimeter-level RTK fixes provide an anchor for the absolute position. When RTK disengages, the network is trained to reproduce ground truth increments as closely as possible. We adopt the Huber Loss [18] because it combines the smooth gradients of the mean squared error (for small residuals) with the outlier tolerance of the mean-absolute error (for large residuals):

(3) $$L_{\sigma }\left(e\right)=\left\{\begin{array}{c} \frac{1}{2}e^{2}\;\;\;\;\;\;\;\;\;\;\;\;\;if\;\left|e\right|\leq \sigma \\ \sigma \left(\left|e\right|-\frac{1}{2}\sigma \right)\;\;\;\;\;\;\;otherwise \end{array}\right.$$

where e is the residual in either the easting or northing channel, and σ=1 m is the switching point. The total GNSS loss is the sum of the horizontal residuals.

(4) $$Loss_{GNSS}={\left|e_{E}\right|}_{\sigma }+{\left|e_{N}\right|}_{\sigma }$$

Weighed by optional bias factors (both set to 1 in this experiment), the Huber formulation prevents large drifts or multipath spikes from dominating gradients while still penalizing fine-scale errors aggressively.

#### 3.3.2. Directional Loss

GNSS increments are vectors; predicting them correctly requires matching the direction as well as magnitude. To isolate the angular component, we compute the bearing at each step and form the wrapped difference:

(5) $$\theta_{p}^{\left(i\right)}=atan2(p_{y}^{\left(i\right)},p_{x}^{\left(i\right)}),{\;\;\;\theta }_{t}^{\left(i\right)}=atan2(t_{y}^{\left(i\right)},t_{x}^{\left(i\right)})$$

(6) $$∆\theta^{i}=atan2(\sin\left(\theta_{pred}^{i}-\theta_{tgt}^{i}\right),cos(\theta_{pred}^{i}-\theta_{tgt}^{i}))$$

(7) $$\delta \theta^{i}=min(\left|∆\theta \right|,\;\;2\pi -|∆\theta |)$$

The mean angular error averages across the look-ahead window and a terminal angular error for only the final angle.

(8) $$L_{mean}=\frac{1}{T}\sum_{i=1}^{T}\delta \theta^{\left(i\right)}$$

(9) $$L_{end}=\delta \theta^{\left(T\right)}$$

The combined directional loss is given by

(10) $$Loss_{Direction}=\alpha \;L_{mean}+\beta \;L_{end}$$

The hyperparameter used is α=0.3 β=0.7. Emphasizing the terminal bearing (70%) reflects our navigation objective: preserving the long-term heading consistency is more critical than small, mid-sequence wiggles.

#### 3.3.3. Vector Loss

Although $L_{GNSS}$ and $L_{Direction}$ constrain the absolute position and orientation, they do not explicitly enforce relative vector fidelity at every anchor. Therefore, a composite Vector Loss is introduced that penalizes discrepancies in both direction (cosine similarity) and residual magnitude.

(11) $$L_{dir}=1-{\hat{p_{i}}}^{T}\hat{t_{i}}$$

where $\hat{p}$ and $\hat{t}$ are the unit vectors calculated from the anchors of the target and predicted increments. Similarly, the magnitude of the vectors is also incorporated. However, they are down weighted to ≤40%, as it teaches to reduce the magnitude loss by decreasing the size of the predicted magnitudes. Concretely putting too much emphasis on the magnitude loss will result in the prediction of very small increments by the network. Magnitude loss is simply the L1 norm of the predicted and target vectors. The final combination is

(12) $$L_{vector}=\lambda_{mag}L_{mag}+\lambda_{dir}L_{dir}$$

where $\lambda_{mag}$ and $\lambda_{dir}$ are the weights for magnitude loss $L_{mag}$ and directional loss $L_{dir}$.

## 4. Experiments and Results

**Figure 4:**
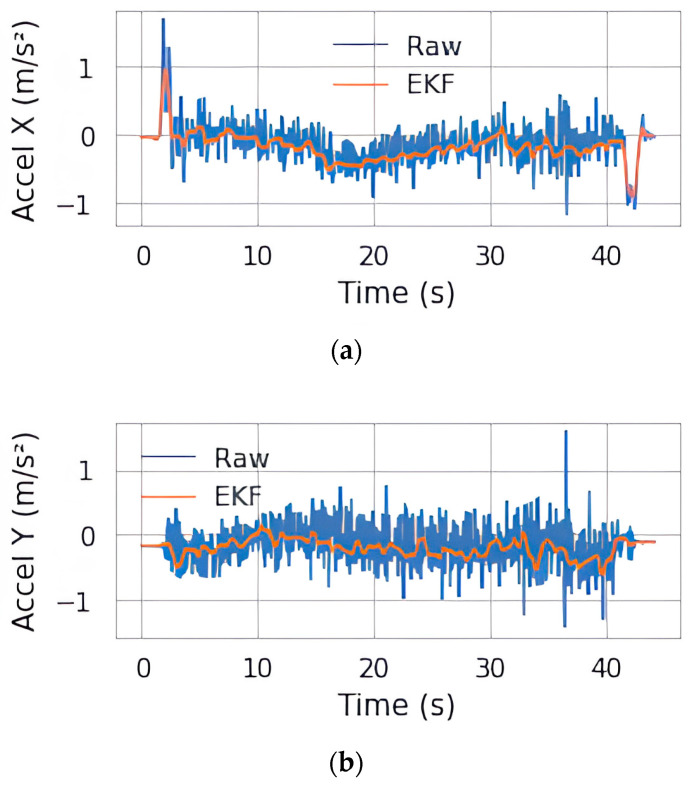
(**a**) Raw and EKF acceleration along the forward direction. (**b**) Raw and EKF acceleration along the lateral direction.

An EKF instance is implemented to reduce noise in acceleration due to vibrations caused by uneven surfaces. This is needed, as the acceleration must match the input velocity information passed to the proposed model for better feature extraction at global and finer levels. The EKF uses a state vector comprising the relative position x and y from the starting position, linear acceleration along the x and y axes, and heading. The state variables in question are the acceleration $a_{x}$ and $a_{y}$, as they are fed to the RTK-SeqNet model. The following set of equations are the part of the update step of the EKF instance:

(13) $$x_{k}=x_{k-1}+rcos(\theta_{k-1}\;)-rcos(\theta_{k-1}+\omega dt)$$

(14) $$y_{k}=y_{k-1}-rsin(\theta_{k-1})+rsin(\theta_{k-1}+\omega dt)$$

(15) $$a_{x,k}=updated\;acceleration\;along\;x$$

(16) $$a_{y,k}=updated\;acceleration\;along\;y$$

(17) $$\theta_{k}=\theta_{k-1}+\omega dt$$

where r is the ratio of linear velocity v with angular velocity ω. It denotes the turning radius of the rover as seen through the input velocities. The rate of change in input velocities is used to calculate acceleration during the prediction step of the EKF algorithm. Figure 4a,b show the raw acceleration signal and the EKF corrected signal along the X (forward) and Y (lateral) direction.

The filter removes the noise in the linear acceleration along both the X and Y direction, clearly showing the movement along the forward direction with no movement (almost constant acceleration) along the lateral direction.

### 4.1. Vector Direction Progression

**Figure 5:**
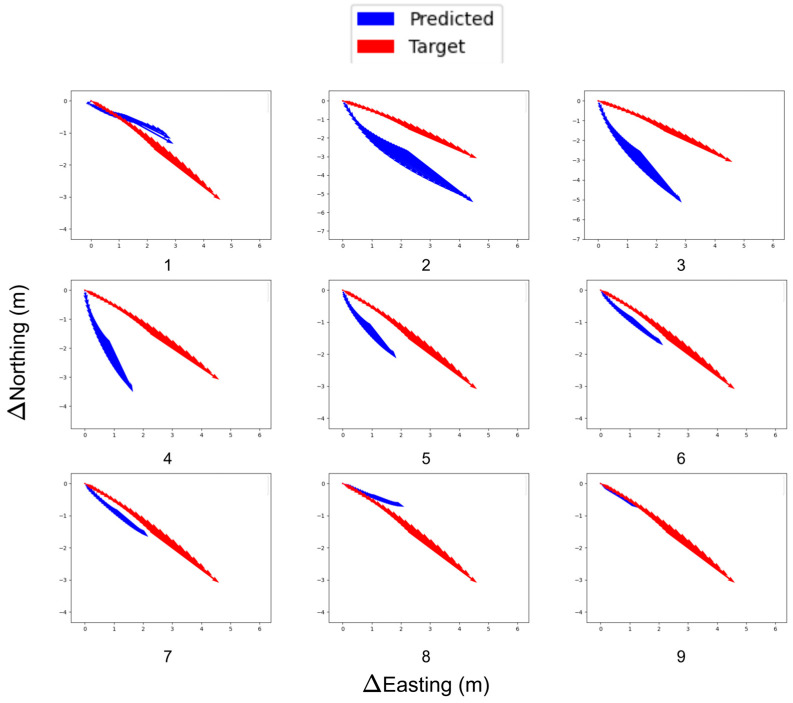
Alignment progression of predicted and target anchors (top-left to bottom-right).

As discussed before, Vector Loss uses cosine similarity to calculate the angle for each anchor vector. These vectors trace the angle progression of the rover over the recent vectors selected for training. Figure 5 shows the change in the normalized vectors’ direction as the training progresses. Evidently, another problem arises where the vector magnitudes diminish as training progresses.

This is inevitable, as the model learns to reduce the training loss by decreasing the magnitude of the vectors. The effects of this problem can be reduced by assigning smaller weights to the magnitudes. The normalized predicted vectors learn to align with the target trajectory as the training progresses.

### 4.2. Model Performance

The proposed RTK-SeqNet model effectively predicts GNSS position increments during signal outages. The results were evaluated using two approaches: a short-interval test and a long-interval test. In the short-interval test, predictions were analyzed over durations of 5–30 s, with performance measured using the average Dynamic Time Warping (DTW) distance [19]. In the long-interval test, predictions were assessed over durations of 5–180 s, and accuracy was quantified using both the DTW and Root Mean Square Error (RMSE).

To evaluate the model’s performance, experiments were conducted where GNSS signal interruptions were simulated by removing the ground truth RTK-enabled GNSS values over varying time durations. The $N_{SEQ}$ number of points that immediately precedes the simulated outages are passed to the model such that an $N_{anc}$ number of future predictions are obtained. As discussed in Section 3.2, since these predictions overlap $N_{anc}$ number of times on the account of being many-to-many as well as operating with a stride of 1, the integration algorithm proposed in Algorithm 1 is used such that the trajectory converges over the predicted timesteps. The DTW and RMSE metrics were utilized to quantify the difference between predicted and actual position increments (Dual EKF with RTK-enabled GNSS), providing a robust measure of prediction accuracy.

**Algorithm 1.** Trajectory Update and Integration1: idx ← 0 2: label_list_copy ← label_list from last iteration 3: Append [0,0] placeholder to label_list 4: **for** each pred in predictions **do** 5:     lbl_idx ← −anchor_size+idx
**do** 6:     **try** 7:         label_list[lbl_idx] ← label_list[lbl_idx − 1]+pred
8:         label_list[lbl_idx] ← (label_list[lbl_idx]+label_list_copy[lbl_idx−1])/2 9:     **except** Index Error **do** 10:        label_list ← predictions[idx:] 11:        break 12:    idx ← idx+1 13: **end for**

**Figure 6:**
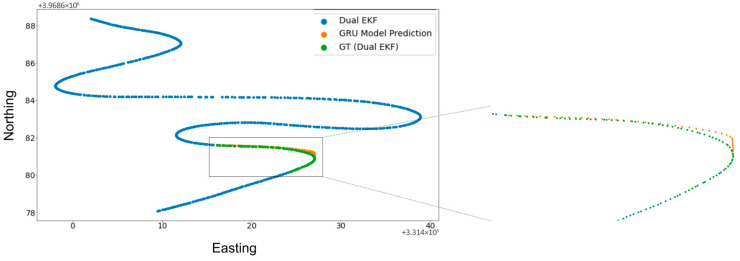
RTK-SeqNet model prediction during a 30 s GNSS outage event (High Speed).

**Figure 7:**
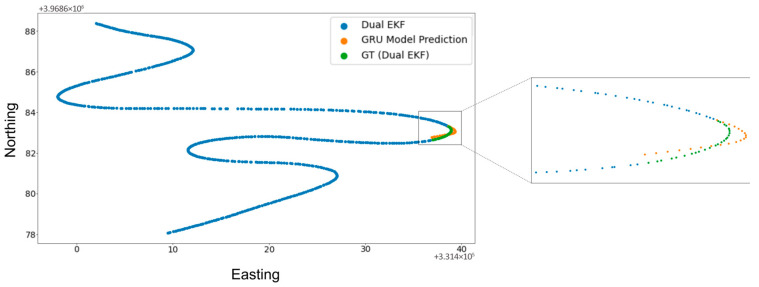
RTK-SeqNet model prediction during a 30 s GNSS outage event (Low Speed).

**Figure 8:**
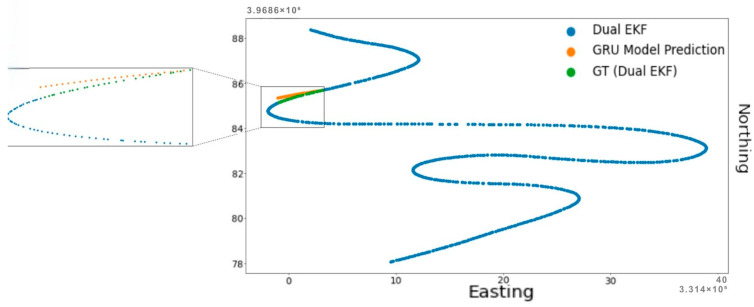
RTK-SeqNet model prediction during a 30 s GNSS outage event (Moderate Speed).

Figure 6, Figure 7 and Figure 8 show the results of the 30 s GNSS outage experiment on High Speed, Low Speed, and Moderate Speed sequences. The speed levels can also be identified by the length of the sequences in question, with the High Speed sequence having the largest length (due to greater inter-point distance) and vice versa. The results show that outages that span a few seconds (30 s in this experiment) show very small differences between the Dual EKF-based GNSS and RTK-SeqNet predicted GNSS position. This suggests that there is more potential for improvement in accuracy if the RTK-SeqNet predicted position is fused with various sensors using the Dual EKF system. This topic is explored in the Discussion section of this paper and is beyond the scope of this study.

**Figure 9:**
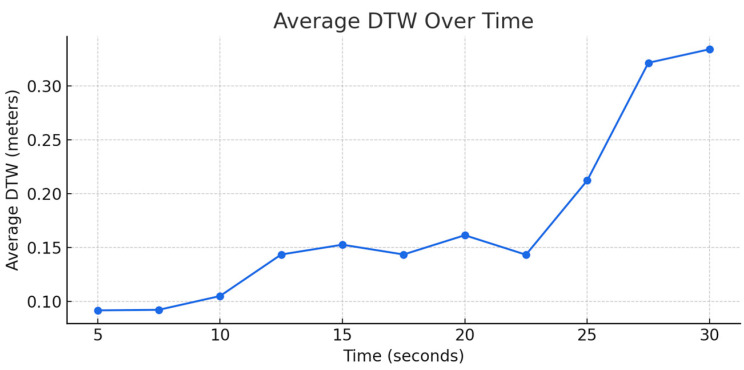
Average DTW over time at High Speed.

**Figure 10:**
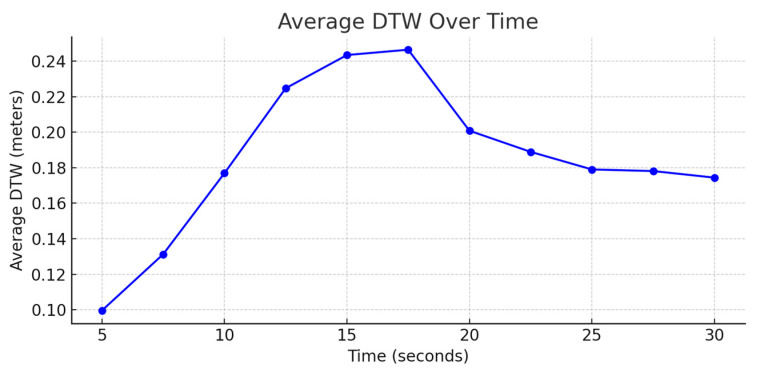
Average DTW over time at Low Speed.

**Figure 11:**
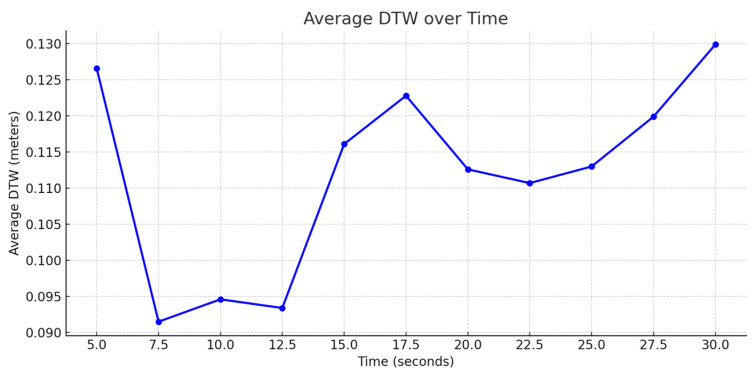
Average DTW over time at Moderate Speed.

The model exhibited a strong performance across a range of prediction intervals: for short prediction intervals, the model maintained consistently high accuracy. As shown in Figure 9, Figure 10 and Figure 11, the model shows below a 25 cm DTW error for the first 20 s of the 30 s test. Beyond the 20 s mark, the error either increases or plateaus. The DTW values were consistently below 0.5 m, indicating that the model’s predictions were highly reliable for short-term GNSS signal outages.

For a linear trajectory at Moderate and High Speeds, the error increases unconstrained; however, in cases of turning points at a Low Speed, the model shows slight convergence. Regardless, it is expected that the error increases overtime, this is because as the GNSS outage time increases, the model predicts future trajectories based on the recent predictions inadvertently accumulating errors. For slower speeds, the inter-point distance is smaller in comparison to higher speeds allowing lower errors.

**Figure 12:**
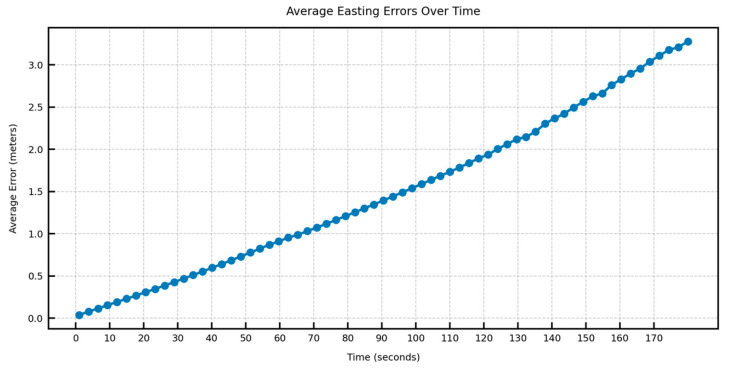
Average easting aDTW over time across the test trajectory.

**Figure 13:**
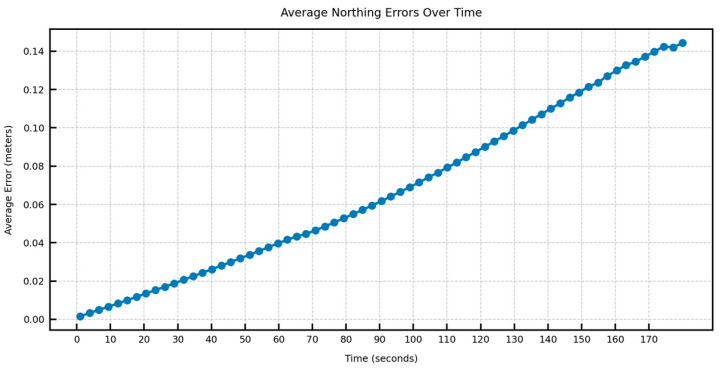
Average northing aDTW over time across the test trajectory.

**Figure 14:**
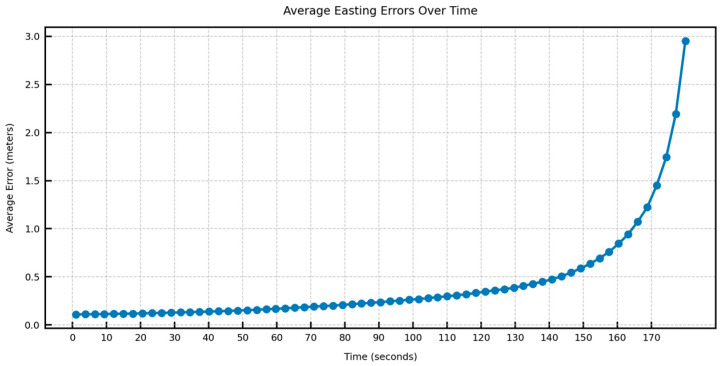
Average easting RMSE over time across the test trajectory.

**Figure 15:**
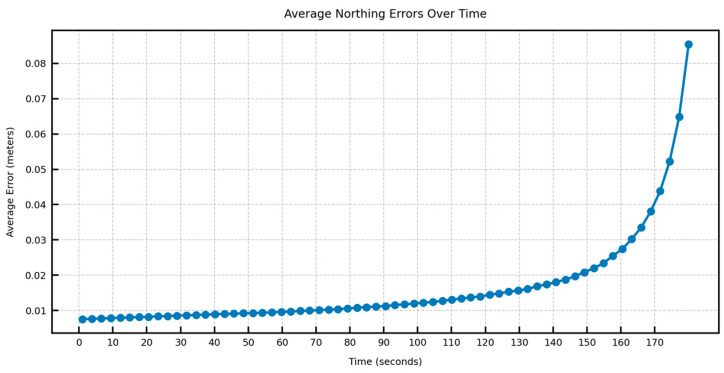
Average northing RMSE over time across the test trajectory.

In Figure 12, Figure 13, Figure 14 and Figure 15, the average DTW and RMSE overtime for the predictions over long trajectories, which in this case is 180 s, averaged across the length of the whole test trajectory, shows a much larger divergence along the easting than the northing. This suggests that the model retains the overall shape of the learned trajectory, but due to mounting errors in the dominant direction (east–west in the test trajectory), the increment errors are also mounted along one direction. This also explains why, within a certain speed limit, the model can converge in terms of error as the direction is changed midway.

Table 5 shows the side-by-side comparison of the average DTW and RMSE of 180 s sequences across the whole test trajectory. The tests are mutually exclusive such that the outages are simulated only for the sequence in question; this is performed for each time period separately. The error at the start is understandably the highest, as the sensor values pick up from zero and require time to converge, which could be a considerable chunk of the allotted 180 s.

## 5. Discussion

Unlike the state-space models that require rigorous parameter tuning and error modeling, a time-series modeling-based approach is much better equipped to model the intricacies of a sequential system. However, it is pertinent to provide the model with accurate directives in terms of objectives so that the most relevant information is learned and prioritized. This paper proposes a weighted combination of objective functions, i.e., GNSS loss for learning predictions deltas, direction loss for detecting changes in the trajectory’s heading prioritizing the later changes, and Vector Loss to scrutinize each anchor direction and the required suitable progression over time such that the overall shape of the trajectory remains relatively intact.

Given that the system utilizes the RTK-enabled GNSS device with a high sampling rate, a many-to-many model is proposed that iteratively averages over the predicted overlapping values, attempting to converge them to the truth values. This approach combined with a multi-level state-space model such as the Dual Extended Kalman Filter approach can greatly enhance the performance of the system. The Dual EKF method employs two Extended Kalman Filter instances running in parallel, i.e., a Local EKF instance and Global EKF instance. Together, these instances compartmentalize complexity, the Local EKF deals with vehicle kinematics (including handling wheel slip via process noise, IMU biases, etc.), and the Global EKF deals with environmental reference data. This compartmentalization simplifies testing and tuning, as each EKF can be verified independently. The Dual Extended Kalman Filter technique is widely used in applications like autonomous navigation, precision agriculture, and UAVs. Levinson et al. utilized dual filter approaches to enhance vehicle localization in urban environments with intermittent GNSS availability [20]. Mahony et al. integrated Dual EKF to achieve precise UAV pose estimation, benefiting from the fast dynamics of aerial platforms [21]. Zhang et al. applied Dual EKF for agricultural machinery navigating fields with partial GNSS coverage, effectively handling sensor outages and drift [22].

If no outage is detected, the GNSS data is converted to odometry information through a navsat_transform module, which then feeds into a Global Extended Kalman Filter (Global EKF) alongside IMU data consisting of body frame accelerations and angular velocities, $f_{ib}^{b}$ and $w_{ib}^{b}$. Global EKF fuses this information to produce a refined GNSS-based position estimate, $P_{GNSS}^{\prime }$, which contributes to the final localization output. Simultaneously, the IMU data also informs a Local EKF, likely tasked with high-frequency local motion estimation. In the event of a GNSS outage, the raw GNSS data is encoded and passed to the proposed Gated Recurrent Unit (GRU)-based network “RTK-SeqNet”, which estimates the GNSS position increment, $∆P_{GNSS}$, thereby providing a predictive odometry output that replaces or supplements the unavailable GNSS input. This output is also integrated into Global EKF to maintain localization continuity. By combining traditional sensor fusion techniques with a recurrent neural network capable of handling temporal dependencies and GNSS dropout, the system achieves robust and accurate localization even under challenging signal conditions.

**Figure 16:**
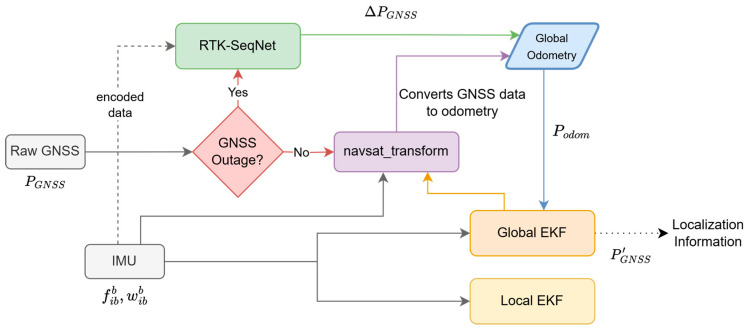
Proposed RTK-SeqNet Network integration with Dual EKF: navsat_transform and the prediction network both publish to a global odometry topic.

The models can be integrated by substituting the GNSS increments as calculated from the RTK GNSS with the increments from the model. As demonstrated, the predicted GNSS increments hold a certain level of accuracy, which can simulate RTK-GNSS during the time when the actual RTK values are unavailable. Figure 16 shows a concrete scheme for hybridization with the popular Dual Extended Kalman Filter algorithm.

## 6. Conclusions

In this paper, we have proposed a novel complementary prediction model to mitigate GNSS outages by predicting RTK-GNSS position increments during signal loss. The model leverages Inertial Measurement Unit (IMU) data combined with temporal sequence learning techniques, including Gated Recurrent Units (GRUs), to provide robust and accurate localization even in environments with unreliable GNSS signals. Experimental results demonstrate the model’s effectiveness, with DTW error metrics below 30 cm for short-term GNSS outages and an average DTW error of 1.6 m and RMSE of 3.4 m for longer trajectories, indicating its potential for real-world applications where GNSS signals are intermittently unavailable.

The integration of this model with a Dual Extended Kalman Filter (Dual EKF) framework shows promise for enhancing the robustness and accuracy of autonomous systems, particularly in precision agriculture and robotic navigation. By providing reliable position corrections when GNSS signals are lost, this approach ensures continuous, precise localization, even in challenging environments. Future work will focus on further refining the hybridization of this model with state-space systems to further improve its performance and adaptability in dynamic conditions. The proposed model represents a significant advancement in autonomous navigation, offering an efficient and scalable solution to the GNSS signal reliability problem.

## Figures and Tables

**Table 1:** Characteristic LSTM and GRU gates and their roles.

Gate	Role (LSTM)	Role (GRU)
Input Gate	Controls how much information flows into the cell state	(Handled by the update gate in GRU)
Forget Gate	Controls how much information is discarded from the cell state	(Also handled by the update gate in GRU)
Output Gate	Controls how much of the cell state is exposed as output	(Not present in GRU)
Update Gate	(Not present in LSTM)	Controls both input and memory retention
Reset Gate	(Not present in LSTM)	Decides how much of the previous state to keep when updating

**Table 2:** Bi-GRU vs. Bi-LSTM parameter and timing comparison.

Recurrent Unit	Parameters	Timing
**Bi-GRU** × 2	810,435	8.545 milliseconds
**Bi-LSTM** × 2	1,008,067	11.14 milliseconds

**Table 3:** Sensor specifications.

Sensor	Sensitivity (Temp Coefficient)	Accuracy/ Stability
Rate Gyroscope	±0.04%/°C	20° drift-s^−1^
Accelerometer	±0.02%/°C	±0.75 mg/°C (X,Y), ±1.5 mg/°C (Z)
Heading	<0.01°	Static pitch/roll ± 1°, dynamic ± 3°; static yaw ± 3°, dynamic ± 5°
GNSS (u-blox ZED-F9P)	0.01 m	-

**Table 4:** Hyper-parameter settings for training the proposed RTK-SeqNet model.

Hyper Parameters	Configuration
Epoch	350
Optimizer	ADAM
Learning Rate	$1\times 10^{-4}$
Weight Decay	$3\times 10^{-3}$
Sequence Length	64
Anchors	32

**Table 5:** Average DTW and RMSE over 180 s segments across the test trajectory.

Trajectories (180 s)	Average DTW	RMSE
0–180 s	2.5953 m	6.5930 m
15–195 s	1.2809 m	3.3427 m
30–210 s	1.5127 m	5.3223 m
50–230 s	1.4278 m	1.4995 m
80–260 s	1.0866 m	1.3645 m
120–300 s	1.6238 m	2.2825 m

## Data Availability

The data presented in this study are available on request from the corresponding author due to its continued use within ongoing research projects.
